# Physical therapy treatment in patients suffering from cervicogenic somatic tinnitus: study protocol for a randomized controlled trial

**DOI:** 10.1186/1745-6215-15-297

**Published:** 2014-07-22

**Authors:** Sarah Michiels, Willem De Hertogh, Steven Truijen, Paul Van de Heyning

**Affiliations:** 1 Department of Rehabilitation Sciences and Physiotherapy, Faculty of Medicine and Health Sciences, University of Antwerp, Antwerp, Belgium; 2 Department of Otorhinolaryngology, Antwerp University Hospital, Edegem, Faculty of Medicine and Health Sciences, University of Antwerp, Antwerp, Belgium; 3 Multidisciplinary Motor Centre Antwerp, University of Antwerp, Antwerp, Belgium; 4 Department of Translational Neurosciences, Faculty of Medicine and Health Sciences, University of Antwerp, Wilrijkstraat 10, 2650 Edegem, Antwerp, Belgium

**Keywords:** Tinnitus, Physical therapy, Treatment, Cervical spine

## Abstract

**Background:**

Tinnitus occurs in a large part of the general population with prevalences ranging from 10% to 15% in an adult population. One subtype is cervicogenic somatic tinnitus, arising from cervical spine dysfunctions, justifying cervical spine assessment and treatment. This study aims to investigate the effect of a standardized physical therapy treatment, directed to the cervical spine, on tinnitus. Additionally, a second aim is to identify a subgroup within the tinnitus population that benefits from physical therapy treatment.

**Methods and design:**

This study is designed as a randomized controlled trial with delayed treatment design. Patients with severe subjective tinnitus (Tinnitus Functional Index (TFI) between 25 and 90 points), in combination with neck complaints (Neck Bournemouth Questionnaire (NBQ) >14 points) will be recruited from the University Hospital of Antwerp.

Patients suffering from tinnitus with clear otological etiologies, severe depression, traumatic cervical spine injury, tumors, cervical spine surgery, or conditions in which physical therapy is contra-indicated, will be excluded.

After screening for eligibility, baseline data such as TFI, NBQ, and a set of cervical biomechanical and sensorimotor tests will be collected.

Patients are randomized in an immediate therapy group and in a group with a delayed start of therapy by 6 weeks.

Patients will receive physical therapy with a maximum of 12 sessions of 30 min for a 6-week program. Data from the TFI and NBQ will be collected at baseline (week 0), at the start of therapy (weeks 0 or 6), at the end of therapy (weeks 6 or 12), 6 weeks after therapy (weeks 12 or 18), and 3 months after therapy (weeks 18 or 24). Secondary outcome measures will be collected at baseline and 6 weeks after the therapy (weeks 12 or 18), as the maximal therapy effect on the cervical spine dysfunctions is expected at that moment.

**Discussion:**

This study is the first to investigate the effect of a standardized physical therapy treatment protocol on somatic tinnitus with a prospective comparative delayed design and with blinded evaluator for baseline, end of therapy, and 6 and 12 weeks after therapy.

**Trial registration:**

12 September 2013, ClinicalTrials.gov: NCT02016313

## Background

Tinnitus is the phantom sensation of sound, in the absence of overt acoustic stimulation [1]. It occurs in 10% to 15% of the adult population [2].

Tinnitus can be related to many different etiologies such as hearing loss, a noise trauma, or the tinnitus may be related to the somatic system of the cervical spine or the temporomandibular area [2].

This study will focus on physical therapy treatment for patients suffering from chronic non-fluctuating subjective cervicogenic somatic tinnitus (CST).

The existence of a link between the cervical spine and tinnitus can be assumed based on several prior studies. Connections between the dorsal column of the spinal cord and the cochlear nuclei (CN) have been found in several animal studies [3,4]. These axons of the dorsal column originate from the C1-C8 dorsal roots of the spinal cord. In particular, stimulation of the C2 dorsal root ganglion generates responses from cells in the CN [5]. Additionally, Matsushima et al. [6] demonstrated that tinnitus improved in 52% of the patients after an occipital nerve block. Other recent studies in humans found that in some patients tinnitus could be evoked or modulated by input from the somatic system, for instance by forceful muscle contractions of the head, neck, and limbs, and pressure on myofacial triggerpoints [7-10]. These findings might explain the ability of some tinnitus patients to modulate their tinnitus by certain head or neck movements. For example, some patients indicate that their tinnitus worsens when performing a combined cervical spine extension and rotation.

Several research groups have investigated the effect of cervical spine treatments on CST [11,12]. Regarding physical therapy treatments, few scientific data are available. Sanchez et al. [11] reviewed five case reports in which cervical spine mobilizations and stretching of suboccipital muscles could decrease the intensity of the tinnitus in five patients. Latifpour et al. [12] showed in a randomized controlled trial (RCT) of 13 tinnitus patients and 11 controls, a greater improvement in tinnitus loudness after application of stretching, posture exercises, and acupuncture compared to controls (*P* >0.001).

Although only case reports and one RCT are reported, the ability of physical therapy treatment directed to the cervical spine to reduce the tinnitus seems promising.

Consequently, the aim of this study is to investigate the effect of a standardized physical therapy treatment program directed to the cervical spine, on several tinnitus and neck related parameters. Additionally, a second aim is to identify a subgroup within the tinnitus population that benefits from the physical therapy treatment.

## Methods

### Patients

Patients will be recruited from the University Hospital of Antwerp by otolaryngologists at their tertiary tinnitus clinic. During this consult, patients will be thoroughly tested to exclude any objective causes of the tinnitus. Patients will be included when suffering from severe chronic non-fluctuating subjective CST, which has been stable for at least 3 months, combined with neck complaints. The severity of the tinnitus will be evaluated using the Tinnitus Functional Index (TFI) [13]. Severe tinnitus is defined as a score between 25 and 90 on the TFI [13]. The presence of a significant neck complaint will be objectified using the 14 points cutoff point of the Neck Bournemouth Questionnaire (NBQ) [14,15]. Patients will be excluded when suffering from objective tinnitus, subjective tinnitus with etiologies, such as hearing loss or Meniere’s disease, severe depression (diagnosed by a psychologist), progressive middle ear pathology, intracranial pathology, traumatic cervical spine injury, tumors, cervical spine surgery, or any cervical spine condition in which physical therapy treatment is contra-indicated. Given the treatment that is studied, patients will also be excluded if they received physical therapy treatment directed to the cervical spine in the past 2 months.

### Study design

**Figure 1:**
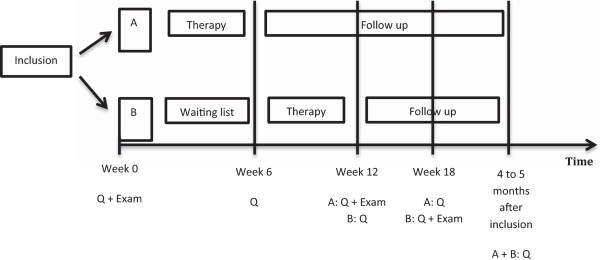
**Delayed treatment design.** Q: Questionnaires. Exam: cervical spine examination.

This study is designed as an RCT to evaluate the effectiveness of a standardized cervical spine treatment on tinnitus and neck-related parameters in patients suffering from CST. A delayed treatment design (Figure 1) will be used to create a waiting list to obtain the data for the control group [16]. At baseline, patients are randomly assigned by the responsible researcher to receive immediate treatment or to the waiting list. In part 1, the immediate treatment group receives the cervical spine treatment for 6 weeks. In part 2, the patients on the waiting list receive cervical spine treatment for the next 6 weeks. The immediate treatment group now enters a 6-week follow-up period. In part 3, all patients enter a follow-up period which ends 12 weeks after the last treatment session.

A pilot study was used for calculating the sample size needed and to optimize the inclusion as well as the follow-up measuring procedure.

The results of the trial will be reported according to the CONSORT guidelines.

### Randomization procedure

After the baseline measurements, the patients are randomized into the immediate treatment group or into the waiting list in a 1:1 ratio based on a block randomization with variable block lengths. The responsible researcher generated a randomization list with Microsoft Excel® software (version 14.3.5, 2010 © Microsoft Corporation). The randomization list is only accessible for the responsible researcher.

### Ethical approval and consent

Ethical approval was obtained from the ethics committee of the University Hospital of Antwerp (reference number: B300201421113). The names of all ethical bodies that approved the study can be found in the additional file. Informed consent is obtained for all patients. The trial is currently in the recruitment phase.

### Outcome measures

The primary outcome measures are the TFI [13] and NBQ [14].

The TFI focuses on eight different domains: the unpleasantness of the tinnitus; reduced sense of control; cognitive interference; sleep disturbance; auditory difficulties attributed to the tinnitus; interference with relaxation; reduction in quality of life; and emotional distress. The test-retest reliability of the TFI is good (r: 0.78). The convergent validity with the Tinnitus Handicap Inventory (r: 0.86) and Visual Analogue Scale (r: 0.75) is good, as well as the discriminant validity with the Beck Depression Inventory-Primary Care (r: 0.56). The clinically relevant reduction was a 13-point reduction.

The pilot study showed that the studied treatment had most influence on the ‘reduced sense of control’ and ‘interference with relaxation’ subscales. Consequently, special attention to these subscales will be paid.

The secondary outcome measures will be the NBQ, a set of different biomechanical and sensorimotor neck parameters and the tinnitus loudness.

The NBQ [14] consists of seven questions on the severity of the neck complaint and its interference with the patient’s wellbeing and professional and daily activities. The test-retest reliability of the NBQ is moderate (ICC: 0.65). The construct validity was acceptable with both the Neck Disability Index (r: 0.50) and the Copenhagen Neck Functional Index (r: 0.44). The effect size was found to be high (Cohen’s d: 1.67), which indicates that the NBQ is highly responsive to changes in cervical spine complaints.

For measuring the biomechanical and sensorimotor neck parameters, the cervical spine mobility will first be investigated in the three cardinal planes.

The range of motion (ROM) is registered using a VICON® measuring device. This device uses the reflection of ultraviolet light on applied markers to perform a 3D movement analysis. The mean absolute error of this measuring device in dynamic conditions is 0.48° (SD 0.05°).

Second, the sensorimotor control of the cervical spine will be measured using the head repositioning accuracy (HRA) to the neutral head position and the continuous linear movement test (CLMT). The HRA measurement will be executed as described by Revel et al. [17] in 1991. The VICON® will be used to register the joint position errors.

The continuous linear movement test (CLMT), as described by Sjolander et al. [18], will be used to objectify the movement speed, acceleration, and Jerk index using the VICON® data. Special attention will be paid to the movement speed and acceleration, as these parameters have proven to have good test-retest reliability [19].

Third, a set of tests will be executed to objectify the impairments in cervical spine mobility and muscle function. This set of tests includes a manual investigation of the cervical spine, tenderness of trigger points, and a strength and endurance test of the deep neck flexor muscles [20]. The manual investigation consists of a manual rotation test and adapted Spurling test [15], and a flexion rotation test evaluating the upper cervical spine rotation mobility [21]. The tenderness of 16 trigger points, is investigated by applying manual pressure [22]. The strength and endurance of the deep neck flexor muscles is objectified using the craniocervical flexion test [20].

All outcome measures will be documented at baseline and at 12 weeks. The primary outcome measures will additionally be documented at 6 and 18 weeks.

### Intervention

**Figure 2:**
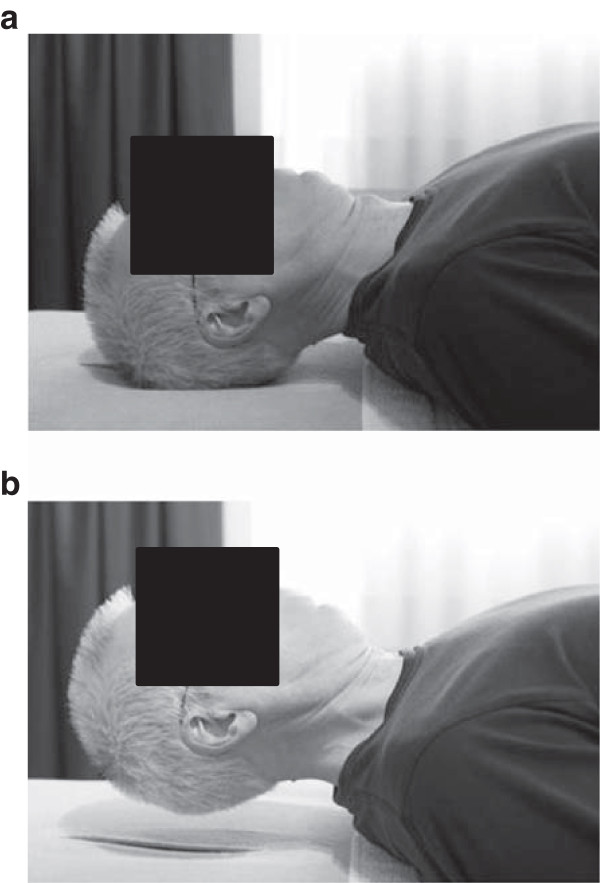
**(a) Craniocervical flexion exercise in supine position (starting position). (b)** Craniocervical flexion exercise in supine position (end position).

**Figure 3:**
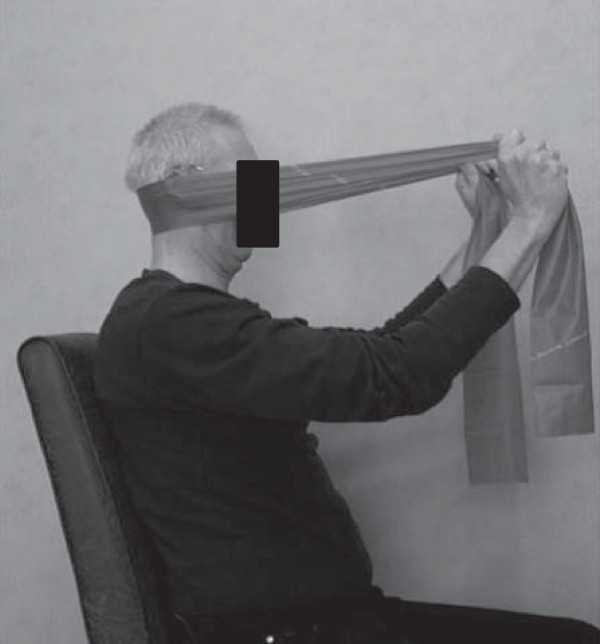
Craniocervical flexion exercise in sitting position.

The intervention, a physical therapy treatment directed to the cervical spine, consists of a multimodal care containing manual mobilizations, exercise therapy, and home exercises. This multimodal physical therapy treatment is based on recent insights of cervical spine therapy [23-25]. Additionally, patients are instructed to perform exercises at home. For these exercises, a booklet established by Castien et al. [26] was adjusted for the tinnitus patients, implementing exercises for the deep neck flexor muscles (Figures 2 and 3) [27] and self-mobilizing exercises [28].

Treatment will be applied by a selected group of physical therapists that all obtained a master’s degree in physical therapy and an additional master’s degree in manual therapy. Moreover, they all participated in a training session organized by the research group. During this training session, the treatment protocol was discussed and trained. Patients included in the trial will be referred for treatment to one of the selected therapists (guided referral). The treatment protocol provides a maximum of 12 standardized physical therapy treatment sessions. The therapists are free to adapt the mobilization techniques and exercises to the current situation of the patient. The therapist registers all performed techniques and exercises.

#### Sample size and power

The sample size was calculated using Medcalc (Medcalc Software bvba.). This calculation was based on data of the primary outcome measure, obtained in a pilot study of 14 patients. Sample size calculation was performed for the clinically relevant change of 13 points in TFI score. The sample size was calculated for the study to have 80% power to reject the null hypothesis (H_0_). The type I error probability, associated with this test, is 0.05. To achieve the 80% power, 17 patients are needed in each group.

The primary analysis population is the intention-to-treat population. This population includes all randomized patients who provided baseline data, regardless of whether or not they adhere to the complete protocol.

#### Statistics

The primary statistical hypotheses are:

H_0_: Change in TFI-baseline to TFI-6 weeks (treated) = Change in TFI-baseline to TFI-6 weeks (waiting list)

Change in NBQ-baseline to NBQ-6 weeks (treated) = Change in NBQ-baseline to NBQ-6 weeks (waiting list)

The primary outcome is a change in the scores on the TFI and NBQ after 6 weeks. The mean change in TFI and NBQ-baseline and TFI and NBQ 6-week follow-up scores will be calculated.

This mean change in TFI and NBQ scores of the treated group will be compared to the mean change in TFI and NBQ scores of the waiting list group.

A repeated measures ANOVA and post hoc tests will be used to compare the mean changes of the treatment and waiting list population at 6 weeks and secondary at baseline, 12, and 18 weeks follow-up.

## Discussion

The aim of this study is to investigate the effect of a standardized physical therapy treatment protocol on several tinnitus and neck-related parameters. Currently, only five case studies and one RCT have studied the effect of physical therapy on somatic tinnitus. Additional RCTs of good quality are therefore needed to verify the effect of physical therapy on somatic tinnitus.

Studies investigating the effect of physical therapy treatment on somatic tinnitus mainly focus on a limited number of treatment modalities. For example, Alcantara et al. [29] and Kessinger et al. [30] focus on manipulations of the cervical spine in individual case studies. Recent studies concerning physical therapy treatment programs directed to the cervical spine, however, indicate that a multimodal treatment, combining mobilization or manipulation and exercise therapy, has better results than a treatment that merely focuses on mobilizations or manipulations [23-25,31].

In this rationale, we decided to use a multimodal treatment in the current study, especially since a prior study from our research group showed a combination of dysfunctions in several structures of the cervical spine in CST patients. These results add to the prospect of a positive effect of a multimodal treatment, since this is directed to multiple dysfunctions at once.

In the current study, all therapists will adjust the treatment modalities to the needs of the individual patient. This pragmatic aspect was chosen to investigate the physical therapy treatment in the full spectrum of everyday clinical settings in order to maximize the applicability and generalizability [32].

This study will need 40 patients with severe tinnitus, also complaining from cervical spine problems. This amount seems feasible, considering the fact that 100 tinnitus patients could be recruited in 6 months in the same setting last year.

The somatic tinnitus population is frequently described as tinnitus that can be modulated by forceful muscle contractions of the head, neck, or limbs [7], or pressure on myofascial trigger points [10]. Based on previous research by Levine et al. [7] and Rocha et al. [10], the inclusion should be limited to the patients who can modulate their tinnitus. As, before inclusion, all objective causes of tinnitus are excluded, we chose to include all patients with a combination of severe subjective tinnitus and neck complaints in order to include all patients who can potentially benefit from our treatment. The patient’s ability to modulate the tinnitus is registered and will be taken into account in the post-treatment analysis.

A control group, receiving no treatment at all, cannot be used in our tertiary referral center due to ethical considerations. Instead, we will use a delayed treatment design to collect the data for the control group. In this type of design, one group of patients will be treated immediately, while the other group will be on the waiting list for 6 weeks and will be treated afterwards.

The pilot study showed that physical therapy treatment has an effect on the TFI scores. Changes in total TFI score however are small. When looking at the different subscales, the largest effect can be noted in the ‘reduced sense of control’ and ‘interference with relaxation’ subscales. To a lesser extent, a change could be noted in the ‘cognitive interference’ subscale. The physical therapy treatment appeared to have little effect on the ‘unpleasantness of the tinnitus’, ‘sleep disturbance’, ‘auditory difficulties attributed to the tinnitus’, ‘reduction in quality of life’, and ‘emotional distress’. Taking into account the content and goals of a physical therapy treatment, the lack of effect on the ‘auditory difficulties attributed to the tinnitus’ seems expectable. Likewise, for a larger effect on the coping related subscales, such as ‘reduction in quality of life’, ‘emotional distress’, and ‘unpleasantness of the tinnitus’, a more psychosocial treatment approach would be necessary.

This study is the first to investigate the effect of a standardized physical therapy treatment protocol on somatic tinnitus with a prospective comparative delayed design and with blinded evaluator for baseline, end of therapy, and 6 and 12 weeks after therapy.

## Consent

Written informed consent was obtained from the patient for publication of this manuscript and accompanying images. A copy of the written consent is available for review by the Editor-in-Chief of this journal.

## Trial status

The study is in the recruitment phase.

## Abbreviations

CN: Cochlear Nuclei; CST: Cervicogenic Somatic Tinnitus; NBQ: Neck Bournemouth Questionnaire; TFI: Tinnitus Functional Index; VAS: Visual Analogue Scale.

## Competing interests

The authors declare that they have no competing interests.

## Authors’ contributions

SM participated in the design of the study and drafted the manuscript. WD conceived of the study, participated in its design and coordination, and helped to draft the manuscript. ST participated in the design of the study and performed the statistical analysis. PV conceived of the study, participated in its design and coordination, and helped to draft the manuscript. All authors read and approved the final manuscript.

## References

[B1] Landgrebe M Azevedo A Baguley D Bauer C Cacace A Coelho C Dornhoffer J Figueiredo R Flor H Hajak G van de Heyning P Hiller W Khedr E Kleinjung T Koller M Lainez JM Londero A Martin WH Mennemeier M Piccirillo J De Ridder D Rupprecht R Searchfield G Vanneste S Zeman F Langguth B Methodological aspects of clinical trials in tinnitus: a proposal for an international standard J Psychosom Res 2012 73 112 121 22789414 10.1016/j.jpsychores.2012.05.002 PMC3897200

[B2] Baguley D McFerran D Hall D Tinnitus Lancet 2013 382 1600 1607 23827090 10.1016/S0140-6736(13)60142-7

[B3] Zhan X Pongstaporn T Ryugo DK Projections of the second cervical dorsal root ganglion to the cochlear nucleus in rats J Comp Neurol 2006 496 335 348 16566003 10.1002/cne.20917 PMC2736115

[B4] Pfaller K Arvidsson J Central distribution of trigeminal and upper cervical primary afferents in the rat studied by anterograde transport of horseradish peroxidase conjugated to wheat germ agglutinin J Comp Neurol 1988 268 91 108 3346387 10.1002/cne.902680110

[B5] Kanold PO Young ED Proprioceptive information from the pinna provides somatosensory input to cat dorsal cochlear nucleus J Neurosci 2001 21 7848 7858 11567076 10.1523/JNEUROSCI.21-19-07848.2001 PMC6762891

[B6] Matsushima JI Sakai N Uemi N Ifukube T Effects of greater occipital nerve block on tinnitus and dizziness Int Tinnitus J 1999 5 40 46 10753418

[B7] Levine RA Hazell J Somatic modulation appears to be a fundamental attribute of tinnitus Proceedings of the Sixth International Tinnitus Seminar 1999 London: The Tinnitus and Hyperacusis Center

[B8] Sanchez TG Guerra GC Lorenzi MC Brandao AL Bento RF The influence of voluntary muscle contractions upon the onset and modulation of tinnitus Audiol Neurootol 2002 7 370 375 12401968 10.1159/000066155

[B9] Sanchez TG da Silva LA Brandao AL Lorenzi MC Bento RF Somatic modulation of tinnitus: test reliability and results after repetitive muscle contraction training Ann Otol Rhinol Laryngol 2007 116 30 35 17305275 10.1177/000348940711600106

[B10] Rocha CA Sanchez TG Myofascial trigger points: another way of modulating tinnitus Prog Brain Res 2007 166 209 214 17956784 10.1016/S0079-6123(07)66018-X

[B11] Sanchez TG Rocha CB Diagnosis and management of somatosensory tinnitus: review article Clinics 2011 66 1089 1094 21808880 10.1590/S1807-59322011000600028 PMC3129953

[B12] Latifpour DH Grenner J Sjodahl C The effect of a new treatment based on somatosensory stimulation in a group of patients with somatically related tinnitus Int Tinnitus J 2009 15 94 99 19842352

[B13] Meikle MB Henry JA Griest SE Stewart BJ Abrams HB McArdle R Myers PJ Newman CW Sandridge S Turk DC Folmer RL Frederick EJ House JW Jacobson GP Kinney SE Martin WH Nagler SM Reich GE Searchfield G Sweetow R Vernon JA The tinnitus functional index: development of a new clinical measure for chronic, intrusive tinnitus Ear Hear 2012 33 153 176 22156949 10.1097/AUD.0b013e31822f67c0

[B14] Bolton JE Humphreys BK The Bournemouth Questionnaire: a short-form comprehensive outcome measure: II Psychometric properties in neck pain patients J Manipulative Physiol Ther 2002 25 141 148 11986574 10.1067/mmt.2002.123333

[B15] De Hertogh WJ Vaes PH Vijverman V De Cordt A Duquet W The clinical examination of neck pain patients: the validity of a group of tests Man Ther 2007 12 50 55 16769236 10.1016/j.math.2006.02.007

[B16] D’Agostino RB Sr The delayed-start study design N Engl J Med 2009 361 1304 1306 19776413 10.1056/NEJMsm0904209

[B17] Revel M Andre-Deshays C Minguet M Cervicocephalic kinesthetic sensibility in patients with cervical pain Arch Phys Med Rehabil 1991 72 288 291 2009044

[B18] Sjolander P Michaelson P Jaric S Djupsjobacka M Sensorimotor disturbances in chronic neck pain–range of motion, peak velocity, smoothness of movement, and repositioning acuity Man Ther 2008 13 122 131 17197230 10.1016/j.math.2006.10.002

[B19] Michiels S Hallemans A Van de Heyning P Truijen S Stassijns G Wuyts F De Hertogh W Measurement of cervical sensorimotor control: The reliability of a continuous linear movement test Man Ther 2014 Mar 7 [Epub ahead of print] 10.1016/j.math.2014.02.004 24656424

[B20] Jull GA O’Leary SP Falla DL Clinical assessment of the deep cervical flexor muscles: the craniocervical flexion test J Manipulative Physiol Ther 2008 31 525 533 18804003 10.1016/j.jmpt.2008.08.003

[B21] Hall T Chan HT Christensen L Odenthal B Wells C Robinson K Efficacy of a C1-C2 self-sustained natural apophyseal glide (SNAG) in the management of cervicogenic headache J Orthop Sports Phys Ther 2007 37 100 107 17416124 10.2519/jospt.2007.2379

[B22] Teachey WS Wijtmans EH Cardarelli F Levine RA Tinnitus of myofascial origin Int Tinnitus J 2012 17 70 73 23906831

[B23] Gross AR Hoving JL Haines TA Goldsmith CH Kay T Aker P Bronfort G Cervical Overview G A Cochrane review of manipulation and mobilization for mechanical neck disorders Spine 2004 29 1541 1548 15247576 10.1097/01.brs.0000131218.35875.ed

[B24] Kay TM Gross A Goldsmith C Santaguida PL Hoving J Bronfort G Cervical Overview G Exercises for mechanical neck disorders Cochrane Database Syst Rev 2012 8 CD004250 16034925 10.1002/14651858.CD004250.pub3

[B25] Miller J Gross A D’Sylva J Burnie SJ Goldsmith CH Graham N Haines T Bronfort G Hoving JL Manual therapy and exercise for neck pain: A systematic review Man Ther 2010 15 334 354 20593537

[B26] Castien RF van der Windt DA Dekker J Mutsaers B Grooten A Effectiveness of manual therapy compared to usual care by the general practitioner for chronic tension-type headache: design of a randomised clinical trial BMC Musculoskelet Disord 2009 10 21 19216763 10.1186/1471-2474-10-21 PMC2662792

[B27] Jull G Trott P Potter H Zito G Niere K Shirley D Emberson J Marschner I Richardson C A randomized controlled trial of exercise and manipulative therapy for cervicogenic headache Spine 2002 27 1835 1843 discussion 1843 12221344 10.1097/00007632-200209010-00004

[B28] Reid SA Rivett DA Katekar MG Callister R Sustained natural apophyseal glides (SNAGs) are an effective treatment for cervicogenic dizziness Man Ther 2008 13 357 366 17951095 10.1016/j.math.2007.03.006

[B29] Alcantara J Plaugher G Klemp DD Salem C Chiropractic care of a patient with temporomandibular disorder and atlas subluxation J Manipulative Physiol Ther 2002 25 63 70 11898020 10.1067/mmt.2002.120415

[B30] Kessinger RC Boneva DV Vertigo, tinnitus, and hearing loss in the geriatric patient J Manipulative Physiol Ther 2000 23 352 362 10863256

[B31] Cleland JA Mintken PE Carpenter K Fritz JM Glynn P Whitman J Childs JD Examination of a clinical prediction rule to identify patients with neck pain likely to benefit from thoracic spine thrust manipulation and a general cervical range of motion exercise: multi-center randomized clinical trial Phys Ther 2010 90 1239 1250 20634268 10.2522/ptj.20100123

[B32] Patsopoulos NA A pragmatic view on pragmatic trials Dialogues Clin Neurosci 2011 13 217 224 21842619 10.31887/DCNS.2011.13.2/npatsopoulos PMC3181997

